# Juvenile vervet monkeys rely on others when responding to danger

**DOI:** 10.1007/s10071-023-01765-2

**Published:** 2023-04-07

**Authors:** Tecla Mohr, Erica van de Waal, Klaus Zuberbühler, Stéphanie Mercier

**Affiliations:** 1 Comparative Cognition Laboratory, University of Neuchâtel, Neuchâtel, Switzerland; 2 Inkawu Vervet Project, Mawana Game Reserve, Swart Mfolozi in Kwazulu-Natal, South Africa; 3 Department of Ecology and Evolution, University of Lausanne, Lausanne, Switzerland; 4 School of Psychology and Neuroscience, University of St Andrews, St Andrews, Scotland, UK; 5 Centre for Functional Biodiversity, School of Life Sciences, University of KwaZulu-Natal, Pietermaritzburg, P/Bag X01, Durban, 3209 South Africa

**Keywords:** Alarm call, Chlorocebus pygerythrus, Audience effect

## Abstract

Primate alarm calls are mainly hardwired but individuals need to adapt their calling behaviours according to the situation. Such learning necessitates recognising locally relevant dangers and may take place via their own experience or by observing others. To investigate monkeys alarm calling behaviour, we carried out a field experiment in which we exposed juvenile vervet monkeys to unfamiliar raptor models in the presence of audiences that differed in experience and reliability. We used audience age as a proxy for experience and relatedness as a proxy for reliability, while quantifying audience reactions to the models. We found a negative correlation between alarm call production and callers’ age. Adults never alarm called, compared to juveniles. We found no overall effect of audience composition and size, with juveniles calling more when with siblings than mothers or unrelated individuals. Finally, concerning audience reactions to the models, we observed juveniles remained silent with vigilant mothers and only alarm called with ignoring mothers, whereas we observed the opposite for siblings: juveniles remained silent with ignoring siblings and called with vigilant siblings. Despite the small sample size, juvenile vervet monkeys, confronted with unfamiliar and potentially dangerous raptors, seem to rely on others to decide whether to alarm call, demonstrating that the choice of the model may play an important key role in the ontogeny of primate alarm call behaviour.

**Supplementary Information:**

The online version contains supplementary material available at 10.1007/s10071-023-01765-2.

## Introduction

How do animals learn to communicate? One influential model stem from research on vervet monkey alarm calls. Adult monkeys were more selective in their alarm call production than juveniles, who responded to a broader range of species, including many non-predators. Presumably, this was the result of a pruning mechanism by which juveniles learned to ignore irrelevant species (Seyfarth and Cheney 1980). Social learning plays a key role (León et al. 2022) but how exactly infants and juveniles obtain the relevant information from others is largely unknown. One key step in successful social learning when learning alarm call is to select appropriate models, i.e., individuals that are competent and reliable. Age and genetic relatedness are likely to be relevant to the choice of the model and there is evidence in meerkats that caller reliability is a relevant feature (Rauber and Manser 2018). Similarly, infant vervet monkeys are more likely to react appropriately to alarm call if they first look at an adult’s reaction compared to others less reliable individuals (Seyfarth and Cheney 1986).

In this study, we were interested in how juvenile vervet monkeys assess their audience during alarm call events. This species is interesting, because adults possess acoustically distinct alarm calls to raptors, terrestrial carnivores and dangerous snakes (Seyfarth et al. 1980), a capacity that develops gradually during ontogeny, as outlined before (Cheney and Seyfarth 1980). To investigate this behaviour process of giving the correct alarm call according to the predator, we presented small unfamiliar raptor models (Fig. S1) to juvenile vervet monkeys (1–2 years old) surrounded by audiences of different compositions, which can be used as a reliable indicator of danger, knowing whether or not to alarm call. For age, we predict that adults will categorise the models as harmless (due to their knowledge and experience) and therefore will not alarm call, while we will expect juveniles to categorise the models as potentially dangerous (due to their resemblance with familiar raptors), and, therefore, to produce alarm calls. Consequently, we will predict juveniles to alarm call in presence of their siblings and their mother but they will remain silent in presence of unrelated conspecifics. Regarding audience size and behaviour, we will predict juveniles to alarm call less in larger than smaller subgroups (due to the likelihood of being surrounded by at least one older group member) and to adapt their alarm call whether mothers and siblings are vigilant assuming that kin would be more trustworthy than non-kin to warn them about danger.

## Methods

The study was conducted over a period of 6 months (30 September 2016–19 March 2017) on three groups of wild vervet monkeys (BD, KB and NH) at the Inkawu Vervet Project (IVP) in Mawana Game Reserve, South Africa (Table S1). Subjects were 15 juveniles (*N* = 9 males; *N* = 6 females; Table S2). We presented unfamiliar raptor models to 15 subjects under three different social conditions (mother, siblings, or unrelated group members), leading to 45 counterbalanced trials. In mother’s condition, we waited until the subject’s mother was within 10 m, making sure that no siblings were present. In the siblings’ condition, we waited until the subject had at least one of his/her siblings present within 10 m, making sure that the mother was absent from the audience. In the unrelated group members ‘condition, we waited until the subject had at least one unrelated conspecific within 10 m, making sure that his/her mother and all siblings were absent. We recorded the reaction as soon as the subject looked in the direction of the model and modified its behaviour (model considered as being detected), which was usually accompanied by vigilance (stopping previous activity and gazing towards model) and/or producing alarm call. Observations finished as soon as the model was covered under a textile. We defined any individual within a 10 m radius of the subject as part of the subject’s audience, which we identified individually and monitored as much as possible in terms of their behaviours. We defined vocal trials as all trials in which at least one alarm call bout had been produced, either by subjects or by other participants.

For each experiment, we collected data on, social condition (mother, siblings, unrelated group members), raptor model (two different ones were used to avoid habituation), subject identity, subject behavioural responses, audience composition (identities of all individuals within 10 m of subject), as well as mother and siblings’ behaviour (ignored, vigilant, alarm called) in our models. For our analysis, we excluded two experiments from the 45 trials, because another individual than the subject had already produced at least one alarm call and could have thus influenced its response. We further removed three trials where the audience reaction was not visible, leading to a total of 40 trials analysed (Table S3). Unfortunately, sample sizes were too small to conduct any statistically meaningful analysis for caller’s age, audience size, age, or reaction. Instead, we present here a descriptive analysis of the main finding. We used generalised linear mixed models (GLMM; Baayen et al. 2008), fitted with a binomial structure and logit-link function with Laplace approximation, to investigate whether juveniles adapted their vocal behaviour according to audience composition (see Supplements: Additional information). Data were analysed with R Studio 3.2.1 (Team 2015). For the GLMM, we used the packages ‘arm’ (Gelman 2016), ‘car’ (Weisberg 2011), ‘faraway’ (Faraway 2016), and lmerTest (Kuznetsova et al. 2017).

## Results

**Fig. 1:**
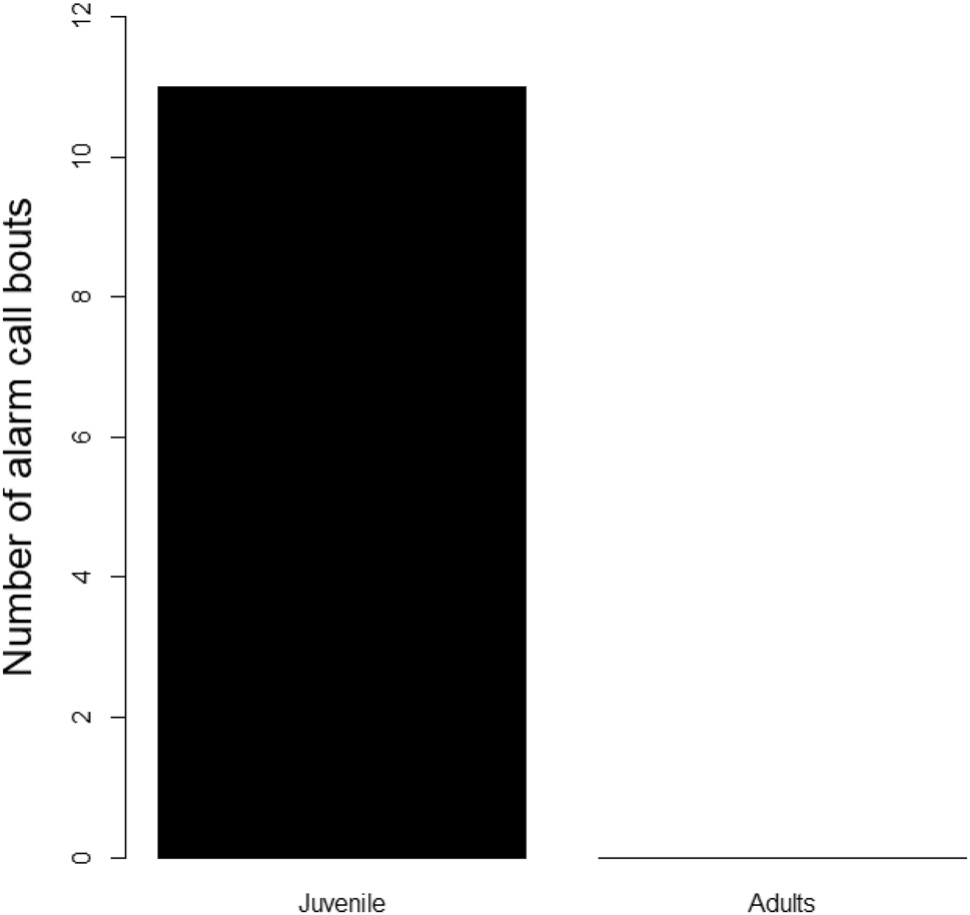
Number of alarm call bouts produced by juveniles and adults

**Fig. 2:**
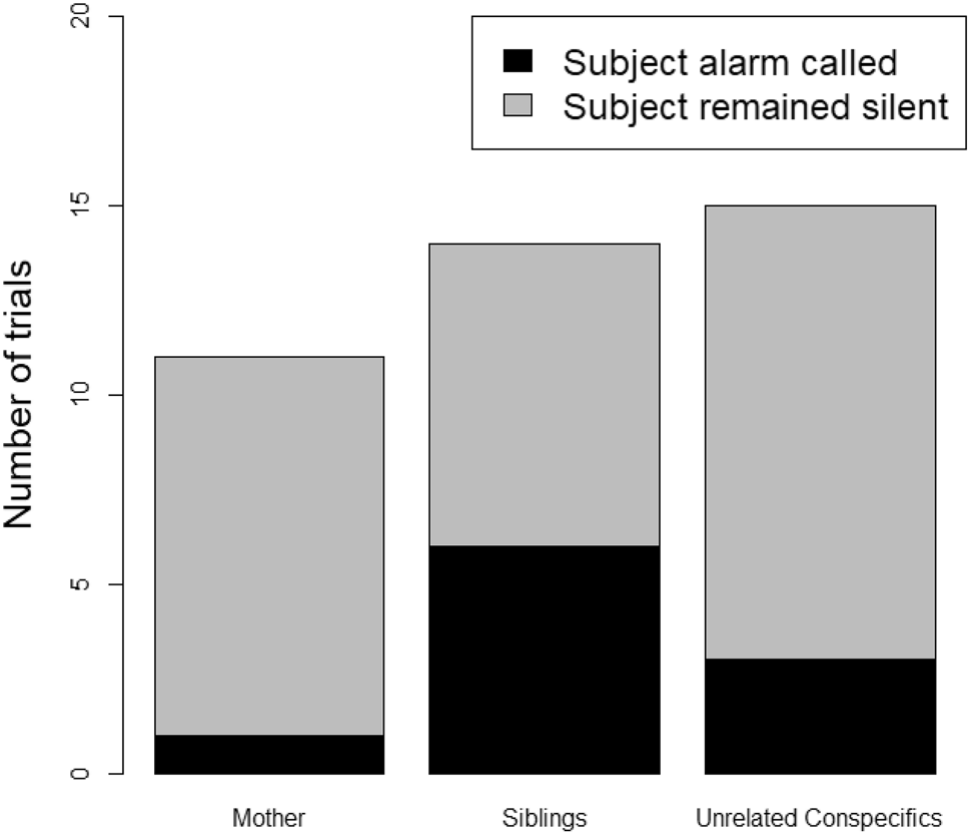
Number of trials in which subjects alarm called (black) or remained silent (grey) in presence of mother, siblings and unrelated conspecifics

*N* = 69 individuals participated in *N* = 40 trials, but only *N* = 9 juveniles alarm called (13.0%, *N* = 11 alarm call bouts total, Fig. 1); with two individuals alarm calling in more than one trial (Table S4). Overall, we found that audience composition did not influence the alarm calling behaviour of juveniles (GLMM, Table S5). However, we observed subjects remaining silent in the presence of their mothers (10 of 11 trials), and to a lesser extent, in the presence of unrelated conspecifics (12 of 15 trials), which was not the case in the presence of siblings (6 of 14 trials; Fig. 2).

**Fig. 3:**
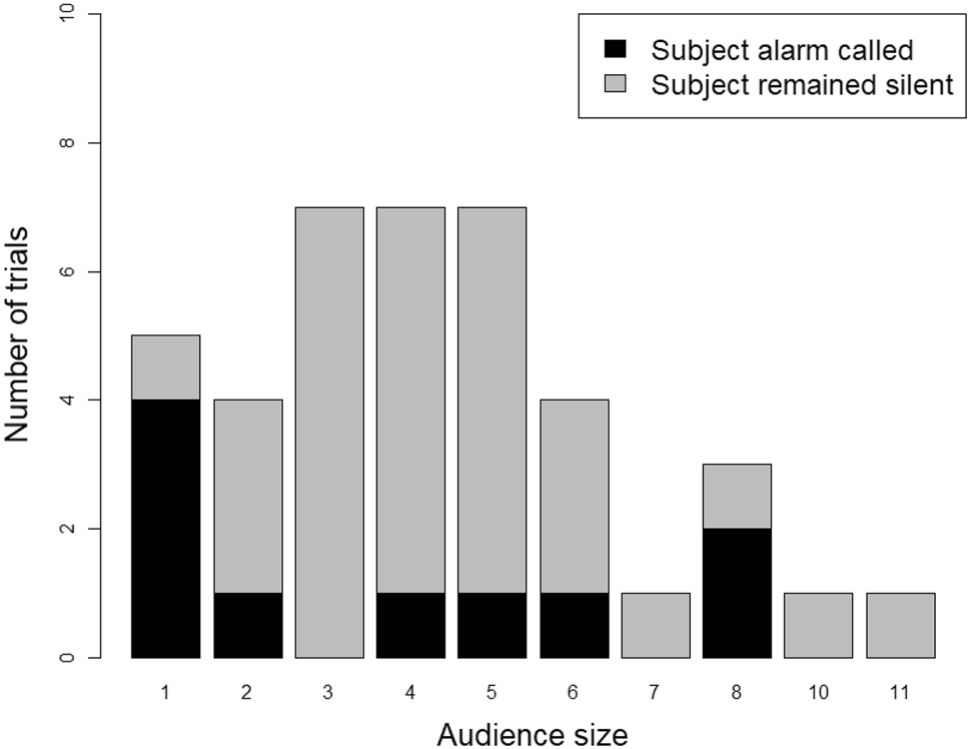
Number of trials in which subjects alarm called (black) and remain silent (grey) in presence of audience size composed of group members from 1 to 11 individuals

We were unable to systematically control for audience size, which ranged from 1 to 11 individuals (Fig. 3).

**Fig. 4:**
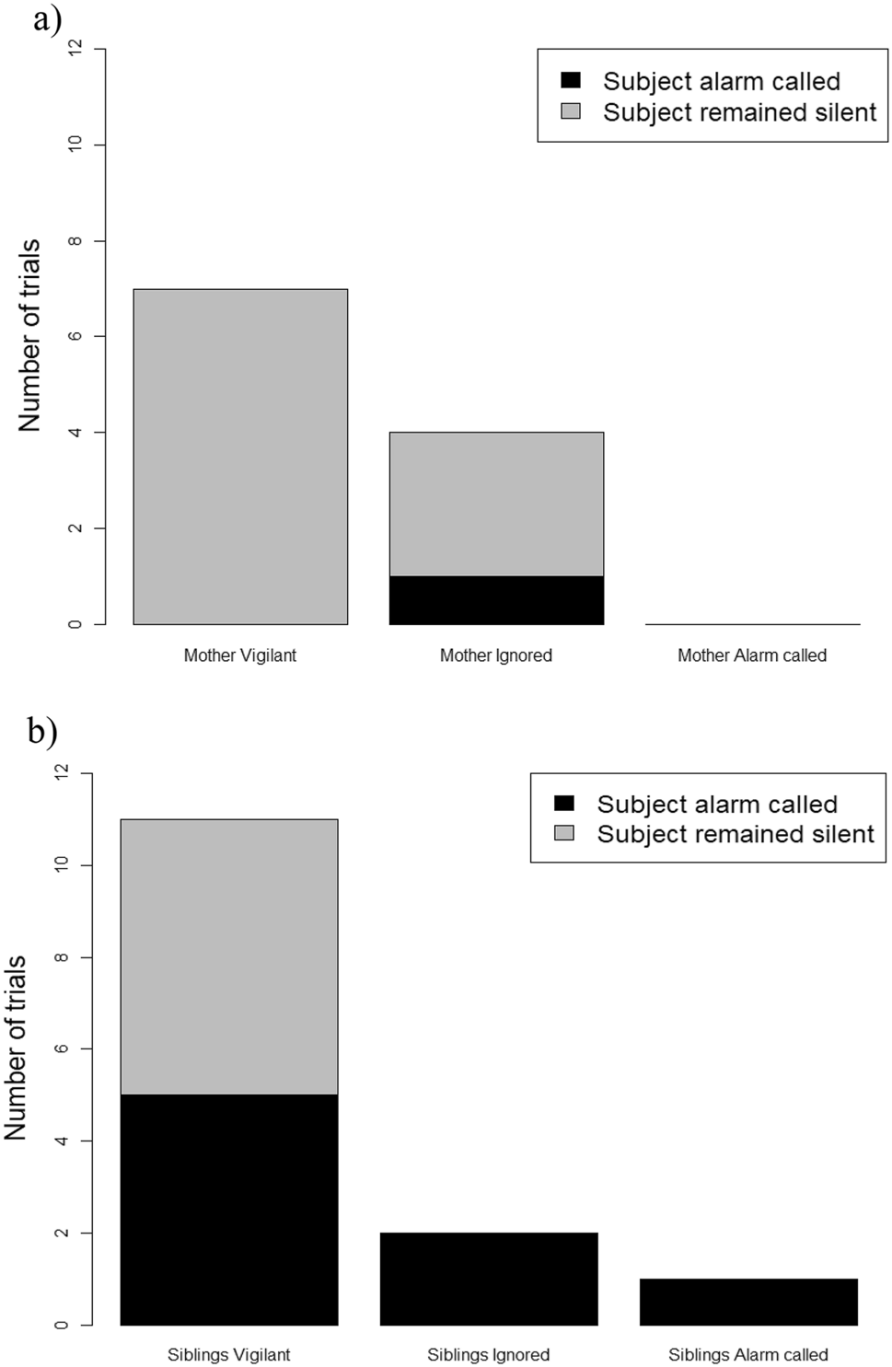
Number of trials in which subjects alarm called (black) and remained silent (grey) in presence of mother (**a**) and siblings (**b**) showing three different reactions to the predator model: vigilant, ignored, alarm called

Regarding the audience reaction, it appeared that whether or not the mother was vigilant may have guided whether the juvenile called (Fig. 4, panel a), whereas the vigilance of siblings did not appear to guide juveniles alarm calling (Fig. 4, panel b).

## Discussion

In this study, we were interested in how juvenile vervet monkeys adapted their anti-predator behaviours when encountering raptor models according to their social environment. First, we found a correlation between caller’s age and alarm call production (Fig. 1), decreasing the production of alarms with age. While adults never alarm called to the models, younger vervet monkeys were more likely to alarm call. This is likely explained by the fact that inexperienced juveniles often alarm called to a wider range of animals, including harmless ones (Seyfarth and Cheney 1980; Wegdell et al. 2019; Wich and de Vries 2006), whereas adults produce alarm calls to specific dangerous known predators.

In a second step, we observed that juveniles were more likely to remain silent in presence of mothers, and to a lesser extent, in presence of unrelated group members, while they were more likely to alarm call in presence of siblings (Fig. 2). Social learning from more experienced individuals could be a potential interesting explanation of why juveniles alarm called less in presence of their mother. Young individuals might benefit from observing the reaction of experienced models to develop more adapted anti-predatory responses.

Finally, regarding audience behaviour, we observed that mother’s vigilance appeared to guide infant alarm calling, but siblings’ vigilance status did not. Subjects alarm called more when the mothers ignored the model, while they remained silent when their mothers were vigilant. This might be explained by a level of awareness since vigilant mothers were clearly aware of the harmless models, while ignoring ones might not be aware of a potential danger nearby. However, we found that juveniles were alarm calling more in presence of siblings who ignored the models, but we did not see any effect in presence of vigilant siblings. Despite the same explanation should be true for siblings than mothers, here, it is possible that results were confounded by kin selection, as it might always be more valuable to alarm call in presence of kin regardless of their awareness, especially if they are younger and thus more vulnerable than the caller. Unfortunately, all our sample sizes are very low and future studies should be conducted to address all these hypotheses with a decent sample size for proper statistical testing.

## Conclusion

In many social animals, alarm calls are essential components of “sometimes-complex” anti-predator strategies, but little is known about how audience and kin selection influence alarm calling behaviours in juveniles. In our project, we found that young vervet monkeys appeared to adjust their alarm calling behaviour depending on the experience in their audience, with increased alarm call production in presence of siblings, compared to when they were with their experienced mothers or unrelated conspecifics. It appears that experience (i.e., age), kinship (mothers and siblings) and specific characteristics of audience (whether or not they are vigilant) all influence the alarm calling of juvenile vervets. Further studies with larger sample sizes are needed to further explore the influences of the social environment in primate vocal development.

## Supplementary Information

Below is the link to the electronic supplementary material.

Supplementary file1 (DOCX 7567 KB)
