# Supplementary material for: Juvenile vervet monkeys rely on others when responding to danger
Source: Anim Cogn. 2023 Apr 7;26(4):1443–7. doi: 10.1007/s10071-023-01765-2 (PMC10344994; doi:10.1007/s10071-023-01765-2)
Supplement: Supplementary file 1 — Supplementary file1 (DOCX 7567 KB) [file 10071_2023_1765_MOESM1_ESM.docx]

**Supplements**

**Fig. S1**. Left panel: picture illustrating the experimental setup with a raptor model positioned on the ground hidden under a brown fabric and an ignorant monkey slowly approaching from behind. Right panel: pictures of both raptor models used in this study.


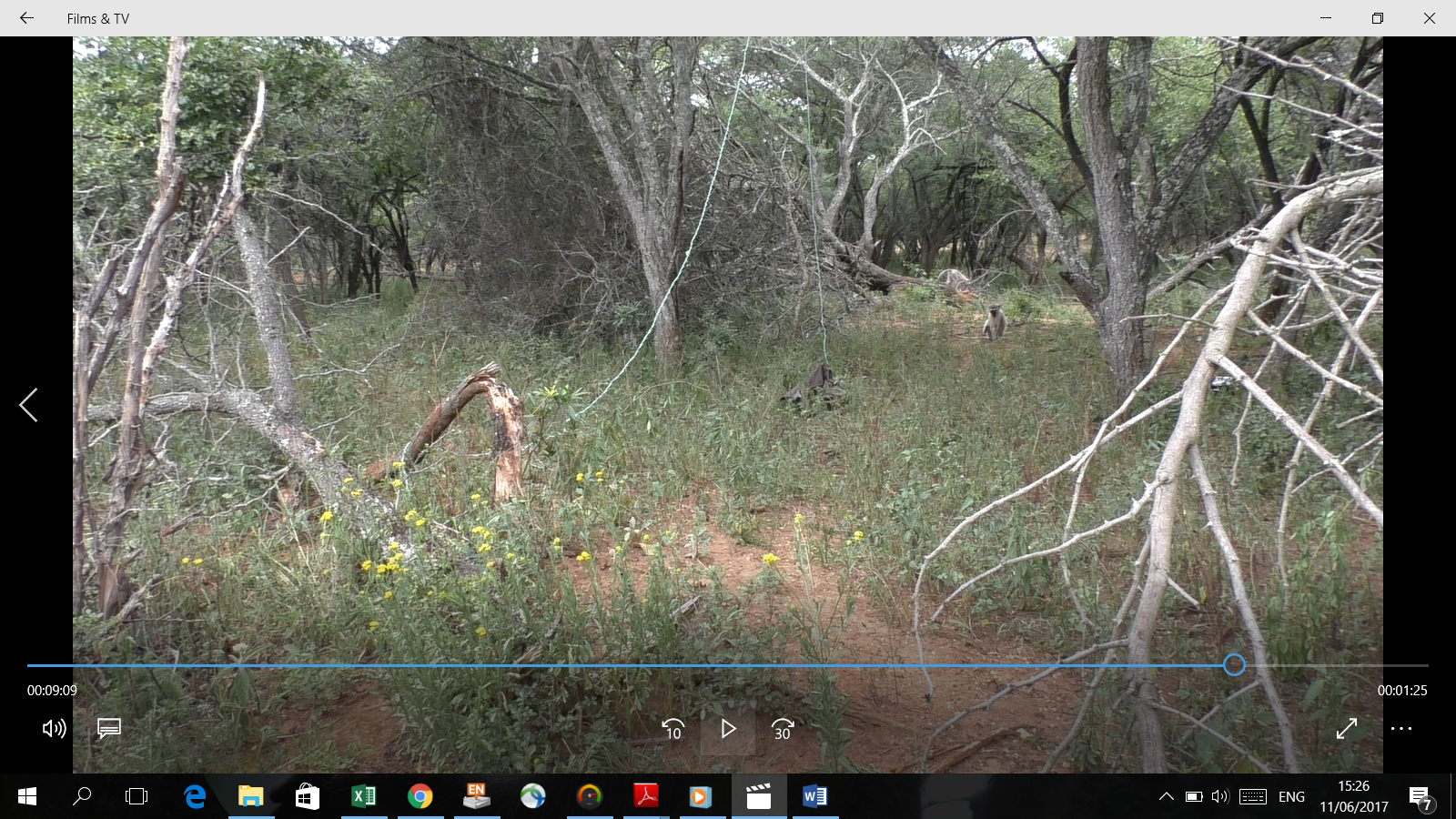

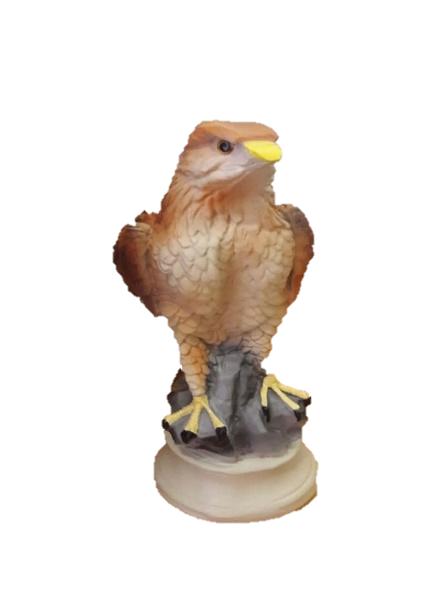

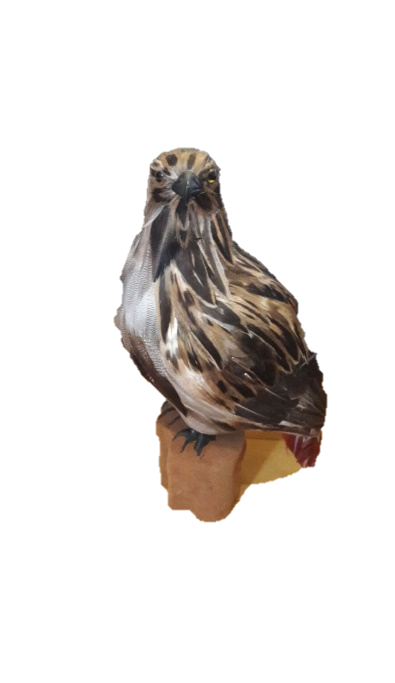


39cm

31cm

**Table. S1**. Composition of the three study groups (BD, KB & NH) at the beginning of the data collection (30 September 2016)

| Group | Adults | Subadults | Juveniles | Infants | Total |
| --- | --- | --- | --- | --- | --- |
| BD | 19 | 10 | 15 | 2 | 46 |
| KB | 7 | 3 | 2 | 5 | 17 |
| NH | 9 | 10 | 10 | 3 | 32 |

| Individuals* | Sex | Date of birth | Age (years) | Group |
| --- | --- | --- | --- | --- |
| Adder | M | 23/11/2015 | 1 | BD |
| Asseblief | M | 05/11/2014 | 2 | BD |
| Granada | F | 08/12/2014 | 2 | NH |
| Heilweis | M | 31/10/2014 | 2 | BD |
| Malawi | M | 14/12/2014 | 2 | KB |
| Nooiens | F | 28/11/2014 | 2 | BD |
| Obelisk | M | 15/11/2014 | 2 | BD |
| Oortjies | F | 20/11/2015 | 1 | BD |
| Prague | M | 19/11/2014 | 2 | NH |
| Pruszkow | M | 23/11/2015 | 1 | NH |
| Polka | F | 28/12/2015 | 1 | BD |
| Ratel | M | 30/11/2015 | 1 | BD |
| Redelik | F | 10/11/2014 | 2 | BD |
| Safari | F | 03/11/2014 | 2 | BD |
| Ulaanbaatar | M | 16/11/2015 | 1 | NH |

**Table. S2.** Description of the 15 individuals used as subjects for the raptor model experiments

**Individuals’ names are further simplified using the following code: three letters for males and four letters for females*

**Table. S3**. Description of the 45 raptor model experiments conducted on 15 subjects

| **Trials** | **Subject** | **Gp** | **Model** | **Subject Al Called** | **Subject was FirstCaller** | **Audience**  **Composition** | **Audience**  **Age** | **Mother**  **Present** | **Mother**  **Reaction** | **Sibling**  **Present** | **Younger Siblings**  **Present** | **Older Siblings**  **Present** | **Siblings**  **Reaction** | **Siblings**  **Al Called** | **Unrelated Conspecific**  **Present** | **Unrelated Present** | **Younger Unrelated**  **Present** | **Older Unrelated**  **Present** | **Unrelated Al Called** | **Audience**  **Size** |
| --- | --- | --- | --- | --- | --- | --- | --- | --- | --- | --- | --- | --- | --- | --- | --- | --- | --- | --- | --- | --- |
| 1 | Add | BD | 1 | Yes | Yes | Unrelated  Conspecific | Older | No | NA | No | NA | NA | NA | NA | Yes | Yes | 0 | 1 | No | 1 |
| 2 | Mal | KB | 1 | Yes | Yes | Siblings | Older | No | NA | Yes | No | Yes | Vigilant | No | No | No | 0 | 0 | No | 1 |
| 3 | Ass | BD | 1 | No | NA | Unrelated  Conspecific | Older | No | NA | No | NA | NA | NA | NA | Yes | Yes | 0 | 2 | No | 2 |
| 4 | Nooi | BD | 1 | Yes | No | Siblings | Older | No | NA | Yes | No | Yes | Vigilant | Yes | No | Yes | 0 | 3 | Yes | 4 |
| 5 | Mal | KB | 1 | No | NA | Unrelated  Conspecific | Mixed | No | NA | No | NA | NA | NA | NA | Yes | Yes | 1 | 3 | No | 4 |
| 6 | Mal | KB | 2 | No | NA | Mother | Older | Yes | Vigilant | No | NA | NA | NA | NA | No | Yes | 0 | 1 | No | 2 |
| 7 | Pol | BD | 2 | Yes | Yes | Siblings | Mixed | No | NA | Yes | No | Yes | Vigilant | No | No | Yes | 2 | 5 | No | 8 |
| 8 | Hei | BD | 2 | No | NA | Unrelated  Conspecific | Mixed | No | NA | No | NA | NA | NA | NA | Yes | Yes | 1 | 5 | No | 6 |
| 9 | Ass | BD | 2 | Yes | Yes | Siblings | Mixed | No | NA | Yes | Yes | Yes | Vigilant | No | No | No | 0 | 0 | No | 2 |
| 10 | Safa | BD | 2 | No | NA | Unrelated  Conspecific | Mixed | No | NA | No | NA | NA | NA | NA | Yes | Yes | 2 | 5 | No | 7 |
| 11 | Ratt | BD | 2 | Yes | Yes | Siblings | Older | No | NA | Yes | No | Yes | Vigilant | No | No | Yes | 0 | 4 | No | 5 |
| 12 | Ratt | BD | 2 | Yes | No | Mother | Mixed | Yes | Ignored | No | NA | NA | NA | NA | No | Yes | 1 | 4 | Yes | 6 |
| 13 | Ass | BD | 2 | Yes | Yes | Mother | Older | Yes | Ignored | No | NA | NA | NA | NA | No | No | 0 | 0 | No | 1 |
| 14 | Pro | NH | 2 | No | NA | Unrelated  Conspecific | Mixed | No | NA | No | NA | NA | NA | NA | Yes | Yes | 1 | 4 | No | 5 |
| 15 | Obe | BD | 2 | No | NA | Unrelated  Conspecific | Older | No | NA | No | NA | NA | NA | NA | Yes | Yes | 0 | 5 | No | 5 |
| 16 | Pro | NH | 2 | Yes | Yes | Siblings | Mixed | No | NA | Yes | Yes | Yes | Vigilant | No | No | Yes | 1 | 2 | No | 4 |
| 17 | Pol | BD | 2 | No | NA | Unrelated  Conspecific | Older | No | NA | No | NA | NA | NA | NA | Yes | Yes | 0 | 3 | Yes | 3 |
| 18 | Rede | BD | 2 | No | NA | Unrelated  Conspecific | Older | No | NA | No | NA | NA | NA | NA | Yes | Yes | 0 | 4 | No | 4 |
| 19 | Pol | BD | 1 | No | NA | Mother | Mixed | Yes | Vigilant | No | NA | NA | NA | NA | No | Yes | 2 | 2 | No | 5 |
| 20 | Pro | NH | 1 | No | NA | Mother | Mixed | Yes | Ignored | No | NA | NA | NA | NA | No | Yes | 2 | 3 | No | 6 |
| 21 | Obe | BD | 1 | Yes | Yes | Siblings | Mixed | No | NA | Yes | Yes | No | Vigilant | No | No | Yes | 5 | 2 | No | 8 |
| 22 | Nooi | BD | 1 | No | NA | Mother | Older | Yes | Ignored | No | NA | NA | NA | NA | No | Yes | 0 | 2 | No | 3 |
| 23 | Safa | BD | 1 | No | NA | Mother | Mixed | Yes | Vigilant | No | NA | NA | NA | NA | No | Yes | 1 | 3 | No | 5 |
| 24 | Ula | NH | 1 | Yes | Yes | Unrelated  Conspecific | Mixed | No | NA | No | NA | NA | NA | NA | Yes | Yes | 3 | 3 | No | 6 |
| 25 | Hei | BD | 1 | No | NA | Siblings | Mixed | No | NA | Yes | No | Yes | Ignored | No | No | Yes | 4 | 3 | No | 8 |
| 26 | Rede | BD | 2 | No | NA | Siblings | Mixed | No | NA | Yes | Yes | Yes | Vigilant | No | No | Yes | 4 | 4 | No | 10 |
| 27 | Ula | NH | 2 | No | NA | Mother | Older | Yes | Vigilant | No | NA | NA | NA | NA | No | Yes | 0 | 4 | No | 5 |
| 28 | Safa | BD | 1 | No | NA | Siblings | Older | No | NA | Yes | No | Yes | Vigilant | No | No | Yes | 0 | 5 | No | 6 |
| 29 | Rede | BD | 1 | No | NA | Mother | Mixed | Yes | Vigilant | No | NA | NA | NA | NA | No | Yes | 1 | 3 | No | 5 |
| 30 | Ula | NH | 2 | No | NA | Siblings | Older | No | NA | Yes | No | Yes | Ignored | No | No | Yes | 0 | 3 | No | 4 |
| 31 | Ratt | BD | 1 | Yes | Yes | Unrelated  Conspecific | Older | No | NA | No | NA | NA | NA | NA | Yes | Yes | 0 | 1 | No | 1 |
| 32 | Oort | BD | 1 | No | NA | Unrelated  Conspecific | Older | No | NA | No | NA | NA | NA | NA | Yes | Yes | 0 | 4 | No | 4 |
| 33 | Gran | NH | 1 | No | NA | Unrelated  Conspecific | Mixed | No | NA | No | NA | NA | NA | NA | Yes | Yes | 1 | 3 | No | 4 |
| 34 | Pru | NH | 1 | No | NA | Mother | Older | Yes | Unk | No | NA | NA | NA | NA | No | Yes | 0 | 4 | No | 5 |
| 35 | Nooi | BD | 2 | No | NA | Unrelated  Conspecific | Younger | No | NA | No | NA | NA | NA | NA | Yes | Yes | 1 | 0 | No | 1 |
| 36 | Oort | BD | 2 | No | NA | Siblings | Older | No | NA | Yes | No | Yes | Vigilant | No | No | Yes | 0 | 2 | No | 3 |
| 37 | Obe | BD | 2 | No | NA | Mother | Younger | Yes | Vigilant | No | NA | NA | NA | NA | No | Yes | 2 | 0 | No | 3 |
| 38 | Hei | BD | 2 | No | NA | Mother | Mixed | Yes | Unk | No | NA | NA | NA | NA | No | Yes | 2 | 2 | No | 5 |
| 39 | Pru | NH | 2 | No | NA | Unrelated  Conspecific | Mixed | No | NA | No | NA | NA | NA | NA | Yes | Yes | 2 | 1 | No | 3 |
| 40 | Pru | NH | 1 | No | NA | Siblings | Older | No | NA | Yes | No | Yes | Vigilant | No | No | Yes | 0 | 2 | No | 3 |
| 41 | Add | BD | 1 | No | NA | Siblings | Older | No | NA | Yes | No | Yes | Vigilant | Yes | No | Yes | 0 | 2 | No | 3 |
| 42 | Add | BD | 2 | No | NA | Mother | Older | Yes | Ignored | No | NA | NA | NA | NA | No | Yes | 0 | 3 | No | 4 |
| 43 | Gran | NH | 2 | No | NA | Siblings | Mixed | No | NA | Yes | No | Yes | Vigilant | No | No | Yes | 6 | 3 | No | 11 |
| 44 | Oort | BD | 2 | No | NA | Mother | Older | Yes | Vigilant | No | NA | NA | NA | NA | No | Yes | 0 | 1 | No | 2 |
| 45 | Gran | NH | 1 | No | NA | Mother | Mixed | Yes | Unk | No | NA | NA | NA | NA | No | Yes | 3 | 3 | No | 7 |

*In grey are the two cases that has been excluded from the analyses since subjects were not the first one to alarm call*

**Table. S4.** Description of all individuals that produced at least one alarm call during at least one trial. Vocal trials thus refer to all experiments in which alarm calls have been produced

| Trials number | Group | Subject | Condition | Caller* | Caller social role | Caller age (years) | Caller  age class |
| --- | --- | --- | --- | --- | --- | --- | --- |
| 1 | BD | Add | Unrelated | Add | Subject | 1 | Juvenile |
| 2 | KB | Mal | Siblings | Mal | Subject | 2 | Juvenile |
| 4 | BD | Nooi | Siblings | Nooi | Subject | 2 | Juvenile |
| 4 | BD | Nooi | Siblings | Nurk | Siblings | 4 | Subadult |
| 4 | BD | Nooi | Siblings | Rak | Unrelated | 4 | Subadult |
| 7 | BD | Pol | Siblings | Pol | Subject | 1 | Juvenile |
| 9 | BD | Ass | Siblings | Ass | Subject | 2 | Juvenile |
| 11 | BD | Ratt | Siblings | Ratt | Subject | 1 | Juvenile |
| 12 | BD | Ratt | Mother | Ratt | Subject | 1 | Juvenile |
| 12 | BD | Ratt | Mother | Nurk | Unrelated | 4 | Subadult |
| 13 | BD | Ass | Mother | Ass | Subject | 2 | Juvenile |
| 16 | NH | Pro | Siblings | Pro | Subject | 2 | Juvenile |
| 17 | BD | Pol | Unrelated | Rak | Unrelated | 4 | Subadult |
| 21 | BD | Obe | Siblings | Obe | Subject | 2 | Juvenile |
| 24 | NH | Ula | Unrelated | Ula | Subject | 2 | Juvenile |
| 31 | BD | Ratt | Unrelated | Ratt | Subject | 1 | Juvenile |
| 41 | BD | Add | Siblings | Ass | Siblings | 2 | Juvenile |

**Since four callers alarm called in several trials (Ass and Ratt called three times while Nurk and Rak called two times), a total of 11 different individuals alarm called during our experiments: nine juveniles and two subadults.*

**Additional information:** General linear mixed model used to test whether juveniles adapted their vocal behaviour according to audience composition

SCNull <- glmer(SubjectAlarmCalled ~ (1|Subject), data=SCdata, family=binomial, control=glmerControl(optimizer="bobyqa"))

SCFullC <- glmer(SubjectAlarmCalled ~ AudienceComposition + (1|Subject), data=SCdata, family=binomial, control=glmerControl(optimizer="bobyqa"))

Subject: Identity of juvenile who was follow before the raptor model.

SubjectAlarmcalled: Whether subject gave an alarm call (Yes or No).

AudienceComposition: The identity of all neighbours present in 2 metres, 5 metres and 10 metres from the subject. Audience were divided in three audience conditions (Mother, Siblings, UnrelatedConspecifics).

**Table. S5.** Results of the GLMM testing subjects’ alarm call production in presence of mothers, siblings and unrelated conspecifics

|  | Estimate | SE | *Z value* | *P-value* |
| --- | --- | --- | --- | --- |
| Intercept | -2.32 | 1.14 | -2.03 | 0.04 |
| Audience composition (Siblings) | 2.02 | 1.23 | 1.64 | 0.10 |
| Audience composition (Unrelated) | 0.92 | 1.24 | 0.74 | 0.46 |

**Table. S6.** Summary table with the identities of callers, subjects’ identities when alarm calls have been produced, subjects vocalisation (Yes or No), condition (Mother, Siblings, Unrelated), parenthetical information (degree of kinship: son, older or younger siblings in relation to the Subject Id) and behaviour of audience (Vigilant, Ignoring, Alarm calling).

| Caller Id | Subject Id | Subject Vocalised | Condition | Parenthetical information | Behaviour audience |
| --- | --- | --- | --- | --- | --- |
| Adder | Adder | Yes | Unrelated | NA | NA |
| Asseblief | Adder | No | Siblings | Older brother | Alarm calling |
| Asseblief | Asseblief | Yes | Siblings | Older and Younger brother | Vigilance |
| Asseblief | Asseblief | Yes | Mother | Son | Ignoring |
| Malawi | Malawi | Yes | Siblings | Younger brother | Vigilance |
| Obelisk | Obelisk | Yes | Siblings | Older brother | Vigilance |
| Poulka | Poulka | Yes | Siblings | Younger sister | Vigilance |
| Propriano | Propriano | Yes | Siblings | Older and Younger brother | Vigilance |
| Rakker | Poulka | No | Unrelated | NA | Alarm calling |
| Rattle | Rattle | Yes | Siblings | Younger brother | Vigilance |
| Rattle | Rattle | Yes | Unrelated | NA | NA |
| Ulambaatar | Ulambaatar | Yes | Unrelated | NA | NA |
